# Management of neck metastases in head and neck cancer: United Kingdom National Multidisciplinary Guidelines

**DOI:** 10.1017/S002221511600058X

**Published:** 2016-05

**Authors:** V Paleri, T G Urbano, H Mehanna, C Repanos, J Lancaster, T Roques, M Patel, M Sen

**Affiliations:** 1 Department of Otolaryngology – Head and Neck Surgery, The Newcastle upon Tyne Hospitals NHS Foundation Trust, Northern Institute of Cancer Research, Newcastle upon Tyne, UK; 2 Department of Oncology, Guy's and St Thomas’ Hospitals, London, UK; 3 Institute of Head and Neck Studies and Education, University of Birmingham, University Hospital, Birmingham, UK; 4 Department of Otolaryngology – Head and Neck Surgery, Queen Alexandra Hospital, Portsmouth, UK; 5 Department of Otolaryngology – Head and Neck Surgery, University Hospital Aintree, Liverpool, UK; 6 Department of Clinical Oncology, Norfolk and Norwich University Hospital, Norwich, UK; 7 Department of Oral and Maxillofacial Surgery, University Hospital of South Manchester NHS Foundation Trust, Manchester, UK; 8 Department of Clinical Oncology, St James's Institute of Oncology, Leeds, UK

## Abstract

This is the official guideline endorsed by the specialty associations involved in the care of head and neck cancer patients in the UK. A rational plan to manage the neck is necessary for all head and neck primaries. With the emergence of new level 1 evidence across several domains of neck metastases, this guideline will identify the evidence-based recommendations for management.

**Recommendations:**

• Computed tomographic or magnetic resonance imaging is mandatory for staging neck disease, with choice of modality dependant on imaging modality used for the primary site, local availability and expertise. (R)

• Patients with a clinically N0 neck, with more than 15–20 per cent risk of occult nodal metastases, should be offered prophylactic treatment of the neck. (R)

• The treatment choice of for the N0 and N+ neck should be guided by the treatment to the primary site. (G)

• If observation is planned for the N0 neck, this should be supplemented by regular ultrasonograms to ensure early detection. (R)

• All patients with T1 and T2 oral cavity cancer and N0 neck should receive prophylactic neck treatment. (R)

• Selective neck dissection (SND) is as effective as modified radical neck dissection for controlling regional disease in N0 necks for all primary sites. (R)

• SND alone is adequate treatment for pN1 neck disease without adverse histological features. (R)

• Post-operative radiation for adverse histologic features following SND confers control rates comparable with more extensive procedures. (R)

• Adjuvant radiation following surgery for patients with adverse histological features improves regional control rates. (R)

• Post-operative chemoradiation improves regional control in patients with extracapsular spread and/or microscopically involved surgical margins. (R)

• Following chemoradiation therapy, complete responders who do not show evidence of active disease on co-registered positron emission tomography–computed tomography (PET–CT) scans performed at 10–12 weeks, do not need salvage neck dissection. (R)

• Salvage surgery should be considered for those with incomplete or equivocal response of nodal disease on PET–CT. (R)

## Introduction

The presence, site and size of metastatic neck disease are important prognostic factors in head and neck squamous cell cancer. Head and neck tumours have a propensity to metastasise to neck nodes and several factors control the natural history and spread of disease. Controversy surrounds the management of the neck in head and neck squamous cell cancer. This is primarily due to the paucity of high-level evidence for many treatment paradigms, but this trend may be reversing with randomised controlled trials and systematic reviews published recently and a few more in progress. This section discusses the management of neck metastases at initial presentation and for residual or recurrent neck disease. It outlines major clinical controversies regarding the management of occult and overt metastatic squamous cell carcinoma (SCC) to the neck nodes.

## Assessment and staging

For the purpose of assessment and documentation, the neck is described in six anatomical levels, (Table I). Level VII is relevant for some head and neck tumours and is included in the table for completeness.

**Table I:** Lymph node levels, sublevels and boundaries

Level	Clinical location	Surgical boundaries	Radiological boundaries
Ia	Submental triangle	S: Symphysis of mandible I: Hyoid bone A (M): Left anterior belly of digastric P (L): Right anterior belly of digastric	Nodes above the level of lower body of hyoid bone, below mylohyoid muscles and anterior to a transverse line drawn through the posterior edge of submandibular gland on an axial image
Ib	Submandibular triangle	S: Body of mandible I: Posterior belly of digastric A (M): Anterior belly of digastric P (L): Stylohyoid muscle	
IIa	Upper jugular	S: Lower level of bony margin of jugular fossa I: Level of lower body of hyoid bone A (M): Stylohyoid muscle P (L): Vertical plane defined by accessory nerve	Superior and inferior limits as described under surgical boundaries Nodes posterior to a transverse plane defined by the posterior surface of submandibular gland and anterior to a transverse line drawn along the posterior border of the sternomastoid. NOTE: Nodes lying medial to the carotids are retropharyngeal and not level II
IIb	Upper jugular	S: Lower level of bony margin of jugular fossa I: Level of lower body of hyoid bone A (M): Vertical plane defined by accessory nerve P (L): Posterior border of sternomastoid muscle	
III	Mid Jugular	S: Level of lower body of hyoid bone I: Horizontal plane along inferior border of anterior cricoid arch A (M): Lateral border of sternohyoid muscle P (L): Posterior border of sternocleidomastoid muscle or sensory branches of the cervical plexus	Superior and inferior limits as described under surgical boundaries Nodes anterior to a transverse line drawn on each axial scan through the posterior edge of the SCM and lateral to the medial margin of the common carotid arteries
IV	Lower jugular	S: Horizontal plane along inferior border of anterior cricoid arch I: Clavicle A (M): Lateral border of sternohyoid muscle P (L): Posterior border of sternocleidomastoid muscle or sensory branches of the cervical plexus	Superior and inferior limits as described under surgical boundaries Nodes anterior to a transverse line drawn on each axial scan through the posterior edge of the SCM and lateral to the medial margin of the common carotid arteries
Va	Posterior triangle	S: Convergence of SCM and trapezius muscles I: Horizontal plane along inferior border of anterior cricoid arch A (M): Posterior border of sternocleidomastoid muscle or sensory branches of the cervical plexus P (L): Anterior border of trapezius muscle	Nodes posterior to a transverse line drawn on each axial scan through the posterior edge of the SCM
Vb	Posterior triangle (supraclavicular)	S: Horizontal plane along inferior border of anterior cricoid arch I: Clavicle A (M): Posterior border of sternocleidomastoid muscle or sensory branches of the cervical plexus. P (L): Anterior border of trapezius muscle	
VI	Anterior compartment	S: Hyoid bone I: Sternal notch A (M): Common carotid artery P (L): Common carotid artery	
VII	Superior mediastinum	S: Sternal notch I: Innominate artery A (M): Common carotid artery P (L): Common carotid artery	

S = superior; I = inferior, A = anterior; P = posterior, L = lateral; M = medial; SCM = sternocleidomastoid

### Clinical palpation

Clinical palpation is regarded as inaccurate (sensitivity and specificity 70–80 per cent) due to factors including inter-operator variability, shape of neck, absence or presence of significant subcutaneous fat and varying size of involved cervical nodes.

### Computed tomographic (CT) and magnetic resonance imaging (MRI) scanning

These techniques have similar sensitivity (81 per cent) in detecting metastatic disease, with CT demonstrating better specificity.^1^ Co-registered positron emission tomography–computed tomography scanning (PET–CT) has been shown to alter initial staging in up to one-third of patients, but the value of this is unclear. This technique has higher sensitivity in picking up clinically occult primaries, synchronous second primaries and distant metastases. PET-CT has demonstrated high negative predictive values in the assessment of neck disease after organ preservation regimes.

### Ultrasound (US) scanning and US-guided fine needle aspiration cytology (FNAC)

Ultrasound has been demonstrated to have consistently high sensitivity (87 per cent) in diagnosing metastatic neck disease. Ultrasound-guided FNAC requires both expertise and experience, and has very high specificity rates (98 per cent) in diagnosis. It should be noted that there are no absolute ultrasound characteristics for differentiating benign from malignant disease.

### Sentinel node biopsy

The aim of this technique is to identify and excise the echelon nodes using radioscintigraphy, which are then tested for occult disease. Patients with no occult disease in the sentinel nodes receive no further treatment for the neck. Meta-analyses suggest that sentinel node biopsy has sensitivity rates exceeding 90 per cent.^2^^,^^3^ A recent prospective multicentre study that recruited 415 patients with 0.5–4 cm transorally resectable SCC and an N0 neck, showed that sentinel node biopsy had a sensitivity, negative predictive value and false negative rate of 86, 95 and 14 per cent, respectively.^4^ Oncological outcomes were not compromised despite only 94 of 415 patients undergoing neck dissection in this cohort.

**Recommendation**

• Computed tomographic or MR imaging is mandatory for staging neck disease, with choice of modality dependent on imaging modality used for the primary site, local availability and expertise (R)

### Neck nodal stage

This should be confirmed and documented in the case record after imaging (certainty factor 2) and prior to treatment planning, using the N category in the 7th edition of the TNM Classification of Malignant Tumours, Union for International Cancer Control (UICC) cancer staging manual. Table II shows the N category to stage neck metastases arising from all head and neck sites excluding those of the nasopharynx, thyroid gland and mucosal melanomas.

**Table II:** Tumour–node–metastasis classification of regional nodes

N_x_	Regional lymph nodes cannot be assessed
N_0_	No regional lymph node metastases
N_1_	Metastasis in a single ipsilateral lymph node 3 cm or less in greatest dimension
N_2_	Metastasis in a single ipsilateral lymph node, more than 3 cm but not more than 6 cm in greatest dimension, or in multiple ipsilateral lymph nodes none more than 6 cm in greatest dimension, or in bilateral or contralateral lymph nodes, none more than 6 cm in greatest dimension
N_2a_	Metastasis in a single ipsilateral lymph node, more than 3 cm but not more than 6 cm in greatest dimension
N_2b_	Metastasis in multiple ipsilateral lymph nodes, none more than 6 cm in greatest dimension
N_2c_	Metastasis in bilateral or contralateral lymph nodes, none more than 6 cm in greatest dimension
N_3_	Metastasis in a lymph node more than 6 cm in greatest dimension

Note: Midline nodes are considered to be ipsilateral nodes

## Treatment options

### Surgery

Historically the mainstay of surgical management of metastatic neck has been neck dissection in its various forms. The standardised neck dissection terminology produced by the American Academy of Otolaryngology and Head and Neck Surgery in 1991 has been updated by the Committee for Neck Dissection Classification of the American Head and Neck Society in 2002^5^ (Table III). There is an increasing trend to divide neck dissections into two broad types with subdivisions: comprehensive (removal of levels I–V) and selective (less than five levels). The need for less extensive surgery in the chemoradiation era, with neck dissection procedures that cannot be classified under the existing systems has led to calls for revision of this system.^6^

**Table III:** Classification of neck dissection techniques

Radical neck dissection (RND)	Removal of levels I–V, accessory nerve, internal jugular vein and sternomastoid muscle
Modified radical neck dissection	Removal of levels I–V dissected; preservation of one or more of the accessory nerve, internal jugular vein or sternomastoid muscle (types I, II, III, respectively)
Selective neck dissection	Preservation of one or more levels of lymph nodes
Extended radical neck dissection	Removal of one or more additional lymphatic and/or non-lymphatic structures(s) relative to a RND, e.g. level VII, retropharyngeal lymph nodes, hypoglossal nerve

It is recommended that the levels or sublevels removed during selective neck dissection (SND) be precisely stated in the operation notes. In order to minimise confusion within labelling the levels during processing, the neck dissection specimen should be divided according to the levels in the operating room and sent to the laboratory in different containers. An alternative is to orientate the neck dissection specimen on a suitable base and label the levels with a marking pen, with or without a photograph, and send it to the laboratory. There is good evidence for reduced long-term morbidity with SND compared with the comprehensive types, namely modified radical neck dissection (MRND) and radical neck dissection (RND). Surgical therapy must be delivered within accredited multidisciplinary teams, by members regularly involved in caring for head and neck cancer patients.

### Radiotherapy

Radiotherapy (RT) should be delivered within an accredited department using megavoltage photons typically from a linear accelerator (typical energy 6 MV). Similar principles should be used for selecting the nodes for RT as are described above for surgery. The probability of microscopic involvement of other nodal groups rises with increasing T-stage and this leads to larger volumes of tissue-requiring irradiation.

Radiotherapy to the neck requires adequate immobilisation and a five-point fixation shell is recommended. Computed tomography  scanning in the treatment position provides the anatomical and electron density information required for RT planning. Conventional and three-dimensional conformal RT often require the use of multiple phases of treatment using photons and electrons of appropriate energy. These techniques have now been superseded by intensity modulated radiotherapy (IMRT), particularly where bilateral nodal irradiation is indicated, where it has been shown to be associated with a reduced risk of late xerostomia and has become the standard of care.

There is now increasing use of concomitant chemoradiotherapy following publication of level 1 studies, suggesting that use of chemoradiotherapy improves overall and progression free survival in advanced head and neck cancer both in the primary and post-operative settings. Altered fractionation regimes have also been shown to offer some advantage over standard fractionation.

## Management strategies for the various neck nodal stages

Treatment of cervical lymph nodes is either elective (in the clinically negative neck) or therapeutic (in the clinically positive neck).

### Management of the clinically node negative neck (N0)

#### New primary

**Fig. 1:**
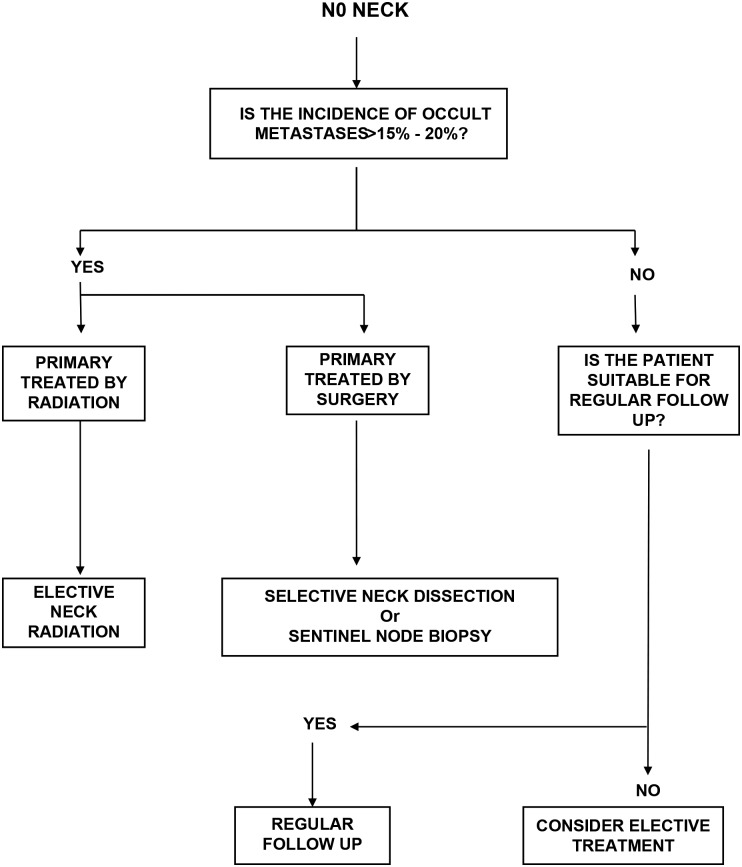
Algorithm for management of the N0 neck.

Clinical and radiological examinations are unable to detect microscopic disease in lymph nodes. Several large retrospective series have reported the incidence of metastases found on histological examination after RNDs in patients with clinically node negative (N0) necks. These figures are useful in identifying the risk of occult metastases in N0 necks and are used to guide clinicians when deciding whether prophylactic treatment of the neck is appropriate (Figure 1).

A study of risk–benefit analysis made in the 1990s using data from retrospective series, when RND was the only procedure widely used for elective neck treatment, suggested that prophylactic treatment of the neck was required if the risk of occult nodal metastases rose above 20 per cent. Given the low morbidity of either available treatment modality, there is support for elective treatment for lesser risk (5–15 per cent). Primary sites with greater than 15 per cent risk of occult metastatic disease in the neck would include almost all squamous cancers of the upper aerodigestive tract except T1 and T2 cancers of the glottis and selected T1 cancers of the oral cavity.

A recent randomised controlled trial (RCT) reported on 500 patients with lateralised stage T1 or T2 oral SCCs randomised to elective neck dissection (*n* = 245) or observation and intervention (*n* = 255), with a median follow up period of 39 months.^7^ At three years, elective node dissection resulted in an improved rate of overall survival (80.0 per cent; 95 per cent confidence interval (CI), 74.1 to 85.8), as compared with therapeutic dissection (67.5 per cent; 95 per cent CI, 61.0 to 73.9), with a hazard ratio for death of 0.64 in the elective-surgery group (95 per cent CI, 0.45 to 0.92; *p* = 0.01 by the log-rank test). Patients in the elective-surgery group also had a higher rate of disease-free survival than those in the therapeutic-surgery group (69.5 per cent *vs* 45.9 per cent, *p* < 0.001). A meta-analysis of all previously published RCTs including data on 283 patients showed that elective neck dissection reduced the risk of disease-specific death (fixed-effects model relative risk = 0.57, 95 per cent CI 0.36–0.89, *p* = 0.014; random-effects model relative risk = 0.59, 95 per cent CI 0.37–0.96, *p* = 0.034) compared with observation.^8^

The classical RND has no role to play in elective treatment of the N0 neck.^9^ The choice lies between an MRND and an SND. Prospective studies suggest SND is as effective as MRND for most primary sites with minimal morbidity. Table IV shows the suggested neck levels that should be addressed for various primary sites, with the recommendations based on a recent analysis of the evidence base.^9^ For oral cavity tumours, SND of levels I to III should be performed. Due to the possibility of skip lesions in level IV, especially in tongue tumours, some studies recommend including level IV. In oropharyngeal, laryngeal and hypopharyngeal tumours, SND of levels II–IV should be performed. Level IIb dissection may not be necessary for the majority of patients, as the incidence of isolated metastasis at this site is less than 2 per cent.^10^

**Table IV:** Recommended neck levels to be dissected for occult neck disease based on primary site

Oral cavity	I–III including IIb
Oropharynx	I–III including IIb; recognise significant chance of contralateral disease
Supraglottis	IIa–III; IIb and IV can be spared. Contralateral SND not indicated for lateralised tumours
Glottis	IIa–III; IIb can be spared. Include IV for T3 and T4 primaries
Subgottis	II–IV, VI
Hypopharynx	II–IV

Elective neck irradiation is as effective as elective neck dissection in controlling subclinical regional disease, with control rates reported to be around 90 per cent. When the primary tumour is treated with RT, first echelon lymph nodes, which are at the greatest risk of harbouring occult disease, are usually included in the high dose or radical RT treatment volume. A large retrospective series comparing elective neck dissection and elective neck irradiation in patients with oral cavity, oropharyngeal and laryngeal cancer reported no statistically significant difference in local control at five years. In patients with hypopharyngeal cancers, local control was significantly better with RT compared with surgery. The consensus guidelines drawn up by experts from clinical research organizations within Europe, Asia, Australia/New Zealand and North America, published in 2014, should be followed for delineation of lymph nodal levels in the node negative neck.^11^

Large retrospective series have reported on the risk of contralateral nodal involvement by each anatomic tumour subsite. As in ipsilateral N0 necks, the contralateral neck should be treated if the estimated risk of occult spread exceeds 15–20 per cent, as occurs with tumours encroaching or crossing the midline. Elective nodal irradiation may be preferred to surgery when both sides of the neck are to be treated.

In long-term follow-up of the untreated N0 neck, consideration should be given where available to ultrasound surveillance and ultrasound-guided aspiration cytology as a method of detecting and treating early disease before it becomes clinically palpable.^12^

#### Recurrent primary cancer

Occult metastatic rates are low (5–10 per cent) in the setting of radiorecurrent cancer if the neck has been included in the radiation field. As neck dissection (ND) in the salvage setting is associated with more complications with no reported benefit, if access to the neck vessels is not needed for primary resection or reconstruction, routine elective neck dissection may not be needed during salvage surgery for locally recurrent primary cancers.

**Recommendations**

• Patients with a clinically N0 neck, with more than 15–20 per cent risk of occult nodal metastases, should be offered prophylactic treatment of the neck (R)
• The treatment choice of the N0 neck should be guided by the treatment to the primary site (G)
• If observation is planned for the N0 neck, this should be supplemented by regular ultrasonograms to ensure early detection (R)
• All patients with T1 and T2 oral cavity cancer and N0 neck should receive prophylactic neck treatment (R)
• Selective neck dissection is effective as MRND for controlling regional disease in N0 necks for all primary sites (R)
• Elective neck dissection and elective neck irradiation have equal efficacy in controlling occult neck disease (R)

### Management of the clinically node positive neck

**Fig. 2:**
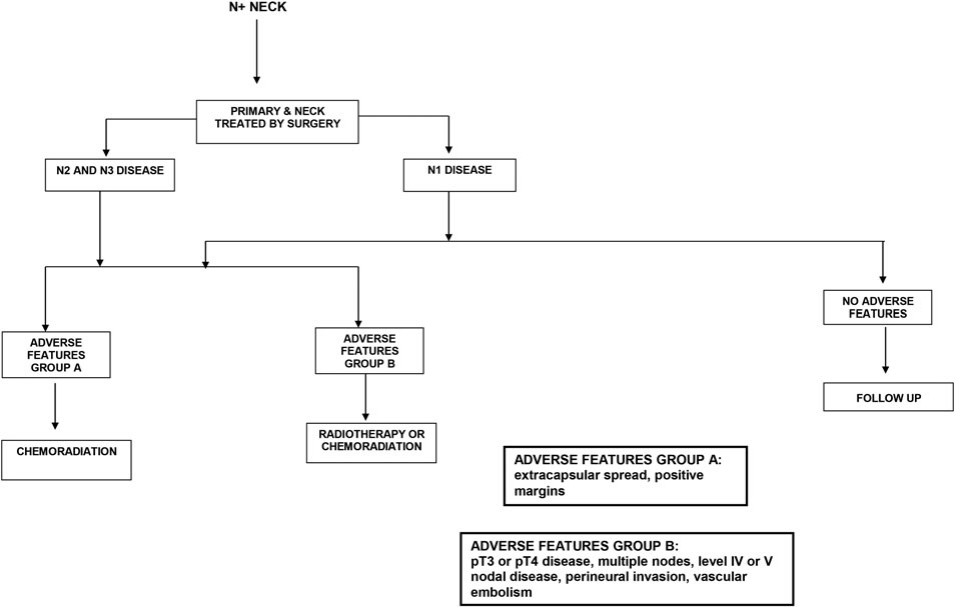
Algorithm for management of the N+ neck when surgery is the primary modality.

**Fig. 3:**
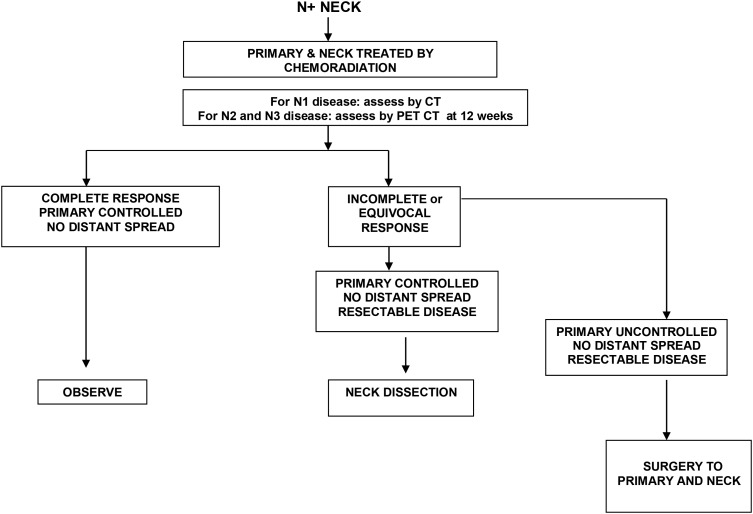
Algorithm for management of the N+ neck when chemoradiation is the primary modality.

When there is clinical or radiological evidence of disease in neck lymph nodes, active treatment is required. Level 1 studies exist to guide the treatment of metastatic neck disease in specific scenarios (Figures 2 and 3). The risk of occult metastases in other apparently uninvolved levels of the neck is high, and depending on the primary site, treatment of these nodes is also required. Level V is least likely to be involved, with between 3 and 7 per cent of patients undergoing RND having positive nodes at level V. The treatment choice of the N+ neck should be guided by the treatment to the primary site, and there is long-term data to support this premise.^13^

#### N1 neck disease

Prospective data from large cancer databases suggest that single modality therapy is sufficient to deal with ipsilateral, single nodes of less than 3 cm in size. If surgery is the chosen modality, SND may be appropriate. As approximately 50 per cent of clinically N1 necks are upstaged after pathological assessment, many patients subsequently require post-operative radiation. Prospective studies have shown that in the absence of bulky disease (N1, N2b), appropriate SND in combination with postoperative RT result in neck control rates equivalent to those achieved by comprehensive neck dissection.^9^ Complete response rates are much higher in patients with nodes of less than 3 cm in size and regional control rates following RT alone are best in patients with nodes less than 2 cm in size.

#### N2 and N3 neck disease

If the primary modality is surgery for this stage of neck disease, MRND and RND result in equivalent rates of disease control in the neck when performed in appropriately selected patients.^9^ Retrospective and prospective studies suggest that adding irradiation post-operatively increases regional control,^14^ especially in the presence of adverse features such as extracapsular nodal spread, positive margins, pT3 or pT4 primary, pN2 or pN3 nodal disease, nodal disease in levels IV or V, peri-neural invasion and vascular invasion. Randomised controlled trials from the EORTC and RTOG have shown improved control with chemoradiotherapy in the post-operative setting, especially in the presence of extracapsular spread and/or microscopically involved surgical margins.^15^ Patients with two or more histopathologically involved lymph nodes without extracapsular spread as their only risk factor did not benefit from the addition of chemotherapy. Morbidity of neck irradiation is higher in patients who have undergone an RND.

If the primary site is suitable for non-surgical treatment, the neck should be treated at the same time. For neck disease staged N2 and above, this will usually involve chemoradiotherapy. The PET-Neck phase III randomised trial compared PET–CT-guided active surveillance with planned neck dissection for neck disease staged N2 or N3 treated by chemoradiotherapy. The study recruited 282 patients into each arm and showed that the survival outcomes were similar with a minimum follow up of two years. A post treatment PET–CT surveillance strategy led to fewer neck dissections, fewer complications, was cost effective (per person cost saving of £1415) and provided 0.07 additional quality adjusted life years compared with planned neck dissection. Based on the results of the PET-Neck trial, there is no role for planned neck dissection after primary chemoradiotherapy.^16^ The current standard of care should be a CT–PET scan between 10 and 12 weeks following chemoradiotherapy, with ND being offered to those who show incomplete or equivocal response of nodal disease. Complete responders may need no further intervention.^17^ The extent of the salvage neck dissection can be based on local protocols, with the recognition that there is an increasing trend to perform a limited neck clearance in these individuals, with removal of the involved level alone or an adjacent level. In patients with fixed and unresectable nodal disease, RT or chemoradiotherapy will be the only options available, but a low likelihood of curative outcome should be recognised.

If the primary tumour is small but sited where resection is not feasible, and associated with advanced neck disease, resection of the nodal disease followed by treatment of the primary tumour by RT (± chemotherapy) plus post-operative RT to the involved neck could potentially be considered but this will be associated with a significant delay in the management of the primary disease which may result in interval primary disease progression.

**Recommendations**

• The treatment choice to the N+ neck should be guided by the treatment to the primary site (G)
• Selective neck dissection alone is adequate treatment for pN1 neck disease without adverse histological features (R)
• Post-operative radiation for adverse histologic features following SND confers control rates comparable to more extensive procedures (R)
• Adjuvant radiation following surgery for patients with adverse histological features improves regional control rates (R)
• Post-operative chemoradiation improves regional control in patients with extracapsular spread and/or microscopically involved surgical margins (R)

## Assessing treatment response

Neck node size and fixity predict response rate and local control with RT alone. In patients with clinical N2 or N3 disease, there is poor correlation between clinical and pathological response following chemoradiotherapy. As discussed above, the PET-Neck trial demonstrated equivalent survival rates to planned neck dissection, with a lower morbidity and a higher overall cost-effectiveness. Co-registered PET–CT scans, performed at least 10 weeks after treatment is now considered the standard of care. A negative PET–CT scan following treatment portends a high disease free survival.^18^ High standard uptake values are associated with residual disease and this can be used to decide the need for neck dissection following primary chemoradiotherapy.^17^^,^^19^^,^^20^

**Recommendations**

• Following chemoradiation therapy, complete responders who do not show evidence of active disease on co-registered PET–CT scans performed at 10–12 weeks, do not need salvage neck dissection (R)
• Salvage surgery should be considered for those with incomplete or equivocal response of nodal disease on PET–CT (R)

## Management of recurrent neck disease

Prior to planning salvage treatment, the patient should be meticulously evaluated for distant metastases. This group is likely to benefit from PET–CT scans to look for distant metastases. If the recurrence has occurred following RT or chemoradiotherapy and is surgically resectable, surgery should be offered but acknowledge the higher risk of complications. In patients who present with unresectable disease, re-irradiation with or without chemotherapy should be considered, particularly in those who present more than two years since their previous treatment. Evidence of partial repair of RT-induced spinal cord subclinical damage and newer RT delivery techniques (IMRT, Tomotherapy^®^, protons) that allow better sparing of neurological, vascular and soft tissue at risk make this a realistic option in a larger number of patients. In patients who recur after previous surgical treatment, options include re-resection followed by adjuvant radiation, or primary RT or chemoradiotherapy.

## Palliative care

Patients who have incurable nodal recurrence present a significant challenge, particularly when distant metastases are not present as people can then live with recurrent disease for many months or longer. Fungating neck nodes have a significant effect on psychosocial function. The impact on speech and swallowing needs careful discussion with dieticians and speech and language therapists so that the potential benefits of tube feeding can be weighed against the risk of over-medicalising terminal care. Specialist palliative care teams should ideally be involved in these discussions before such complications develop.

There may be occasions where palliative RT, chemotherapy or surgery have the potential to improve quality of life (QoL) in this situation. The overall expected prognosis, patient perspective and goals, morbidity of treatment and likely benefits need to be openly discussed to ensure that there is a reasonable expectation that any intervention will improve QoL for a given individual.

## Ongoing research

Current portfolio studies open to recruitment and relevant to neck metastases include: the role of SND in patients with early oral SCC (1–3 cm primary size) and no clinical evidence of lymph node metastases in the neck (SEND trial).

### Key points

• The neck stage is the single most important tumour prognostic factor
• Prognosis is affected by number of involved nodes, the anatomic level in the neck, tumour load, the presence of extracapsular spread, peri-neural and vascular invasion, previous treatment by surgery or radiotherapy and resectability
• A large number of malignant nodes will measure less than 10 mm in diameter and extracapsular spread will occur in a substantial percentage of smaller nodes, as small as 2 mm. These may not be identified on conventional (CT and magnetic resonance) imaging
• Incidence of nodal metastases depends on site and size of the primary tumour. This figure may be as low as 1 per cent for early glottic tumours or as high as 80 per cent for nasopharyngeal carcinomas
• The majority of tumours will metastasise in a predictable manner to certain nodal groups but it should be remembered that tumours can metastasise to more remote sites (i.e. nasopharyngeal cancers to level V, tongue cancers to level IV) and that the pattern of spread will be disrupted by previous surgery or radiotherapy
• The possibility of bilateral nodal disease should be considered especially when the primary site involves the tongue base, nasopharynx or supraglottic larynx or when the primary site crosses midline
• Neck dissections should be documented as per the accepted classification system
• Radiotherapy target delineation should follow the internationally recognised consensus guidelines
• Standardised reporting of neck dissection specimens according to the Royal College of Pathologists data set is essential
• Issues of function and quality of life have to be considered in the management of metastatic neck disease.
